# The Association of Socioeconomic Status and Access to Low-Volume Service Providers in Breast Cancer

**DOI:** 10.1371/journal.pone.0081801

**Published:** 2013-12-02

**Authors:** Chun-Ming Chang, Wen-Yao Yin, Chang-Kuo Wei, Chun-Hung Lin, Kuang-Yung Huang, Shih-Pin Lin, Cheng-Hung Lee, Pesus Chou, Ching-Chih Lee

**Affiliations:** 1 Department of Surgery, Buddhist Dalin Tzu Chi General Hospital, Chiayi, Taiwan; 2 Department of Otolaryngology, Buddhist Dalin Tzu Chi General Hospital, Chiayi, Taiwan; 3 Center for Clinical Epidemiology and Biostatistics, Buddhist Dalin Tzu Chi General Hospital, Chiayi, Taiwan; 4 Division of Rheumatology, Department of Internal Medicine, Buddhist Dalin Tzu Chi General Hospital, Chiayi, Taiwan; 5 Community Medicine Research Center and Institute of Public Health, National Yang-Ming University, Taipei, Taiwan; 6 School of Medicine, Tzu Chi University, Hualian, Taiwan; Boston Children's Hospital, United States of America

## Abstract

**Background:**

No large-scale study has explored the combined effect of patients’ individual and neighborhood socioeconomic status (SES) on their access to a low-volume provider for breast cancer surgery. The purpose of this study was to explore under a nationwide universal health insurance system whether breast cancer patients from a lower individual and neighborhood SES are disproportionately receiving breast cancer surgery from low-volume providers.

**Methods:**

5,750 patients who underwent breast cancer surgery in 2006 were identified from the Taiwan National Health Insurance Research Database. The Cox proportional hazards model was used to compare the access to a low-volume provider between the different individual and neighborhood SES groups after adjusting for possible confounding and risk factors. Hosmer-Lemeshow goodness-of-fit statistic was used to determine how well the model fit the data.

**Results:**

Univariate analysis data shows that patients in disadvantaged neighborhood were more likely to receive breast cancer surgery at low-volume hospitals; and lower-SES patients were more likely to receive surgery from low-volume surgeons. In multivariate analysis, after adjusting for patient characteristics, the odds ratios of moderate- and low-SES patients in disadvantaged neighborhood receiving surgery at low-volume hospitals was 1.47 (95% confidence interval=1.19-1.81) and 1.31 (95% confidence interval=1.05-1.64) respectively compared with high-SES patients in advantaged neighborhood. Moderate- and low-SES patients from either advantaged or disadvantaged neighborhood had an odds ratios ranging from 1.51 to 1.80 (p<0.001) to receiving surgery from low-volume surgeons. In Hosmer-Lemeshow goodness-of-fit test, p>0.05 that shows the model has a good fit.

**Conclusions:**

In this population-based cross-sectional study, even under a nationwide universal health insurance system, disparities in access to healthcare existed. Breast cancer patients from a lower individual and neighborhood SES are more likely to receive breast cancer surgery from low-volume providers. The authorities and public health policies should keep focusing on these vulnerable groups.

## Introduction

Previous studies have showed that breast cancer patients treated by low volume providers have inferior outcomes and survival than those treated by high volume providers [1,2]. Several studies have revealed predictors for low-volume providers among cancer patients. Among these factors, socioeconomic status was an important determinant. Several studies have showed that breast cancer patients of low SES, living in rural areas, far away from high volume hospitals were more likely to be treated at low-volume hospitals and by low-volume surgeons [3,4]. Low-SES patients might have less access to a high-volume provider due to their insurance status and live far away from a high volume hospital, which might be another important reason for survival disparities in breast cancer. However, most studies explore the association of individual SES and the access to a low-volume provider on a hospital or surgeon level [3–5]. Also, no large-scale study has explored the combined effect of patients’ individual and neighborhood SES on their access to a low-volume provider for breast cancer surgery. Moreover, it has not been fully understood that under a nationwide universal health insurance system, patients are able to seek care with any hospital or physician, how significant the breast cancer patients’ individual and neighborhood SES influences their choices on where to undergo breast cancer surgery. 

The purpose of this study was to explore whether breast cancer patients from a lower individual and neighborhood SES are disproportionately receiving breast cancer surgery from low-volume providers under a nationwide universal health insurance system by using the Taiwan National Health Insurance Research Database (NHIRD).

## Materials and Methods

### Ethics Statement

This study was initiated after approval by the Institutional Review Board of Buddhist Dalin Tzu Chi General Hospital, Taiwan (IRB B1000101). Since all identifying personal information was removed from the secondary files prior to analysis, the review board waived the requirement for written informed consent from the patients involved.

### Database

We used data from 2006 from the National Health Insurance (NHI) Research Database, which covered medical benefit claims for over 23 million people in Taiwan (approximately 97 percent of the island’s population). These databases were monitored for completeness and accuracy by Taiwan’s Department of Health. Taiwan’s NHI has the unique characteristics of universal insurance coverage and a single-payer system with the government as sole insurer. Patients have free access to seek care with any physician or hospital they choose. 

Patients with breast cancer in Taiwan who underwent breast cancer surgery (modified radical mastectomy, simple mastectomy, and partial mastectomy) in 2006 were identified from the database and included in this study. The NHI research database identified 5,750 patients receiving breast cancer surgery in 2006. 

### Definition of low-volume providers

The method for defining high- and low-hospital volume and surgeon volume of breast cancer surgery was: (1) Hospitals were categorized by their total patient volume by using unique hospital identifiers in this database. The 5,750 patients were sorted into three approximately equal groups based on hospital volvolume: 87 cases (low), 91-216 cases (medium), and 217-456 cases (high) (Table S1 in Appendix S1) (2). Surgeons were categorized by their total patient volume by using unique identifiers in this database. These patients were sorted into three approximately equal groups based on surgeon volvolume: 20 cases (low), 21-80 cases (medium), and 88-217 cases (high) (Table S2 in Appendix S1) (3). We merged high- and medium-volume hospital/surgeon as high-volume hospital/surgeon because of their similar survival outcomes, which was demonstrated in our previous study [2]. The low-volume hospital and surgeon group were the low-volume providers in the study. 

### Individual-level Measures

The key independent variables of the current study were the interaction effects of individual SES and neighborhood SES on selection of low-volume providers. Patient characteristics included age, geographic location, severity of disease, and SES. The disease severity of each patient was based on the modified Charlson Comorbidity Index Score (CCIS), which has been widely accepted in recent years for risk adjustment in administrative claims data sets [6].

This study used income-related insurance payment amount as a proxy measure of individual SES, which is an important prognostic factor for cancer [7–9]. The breast cancer patients were classified into three groups: (1) low SES, lower than US$528 per month (New Taiwan Dollars (NT) 0, $1 to $15,840), (2) moderate SES, between US$528 to $833 per month (NT $15,841 to $25,000), and (3) high SES, US$833 per month (NT $25,001) or more [9]. We selected NT$15,840 as the low income level cutoff point because this was the government-stipulated minimum wage for full-time employees in Taiwan in 2006. 

### Neighborhood-level socioeconomic status

Neighborhood SES is a contextual factor based on neighborhood household income averages and percentages reported in Taiwan’s Census. In that census, neighborhood household income was measured by township using per capita income which could be determined based on tax statistics released by Taiwan’s Ministry of Finance, (http://www.fdc.gov.tw/dp.asp?mp=5). The categorization into advantaged or disadvantaged neighborhoods was based on the median values, with advantaged neighborhoods having higher-than-median neighborhood household incomes and disadvantaged neighborhoods having lower-than-median household incomes.

### Statistical Analysis

The SPSS (version 15, SPSS Inc., Chicago, IL, USA) were used to analyze data. A p-value of <0.05 was used to determine statistical significance. Univariate associations were evaluated by Pearson’s chi-square test. Multiple logistic regression was used to estimate the adjusted odds ratio for receiving breast cancer surgery based on each patient’s observed variables, which included age, CCIS, individual and neighborhood SES, and urbanization and regions of residence. Hosmer-Lemeshow goodness-of-fit test was used to determine how well the model fit the data.

## Results

A total of 5,750 patients who received breast cancer surgery were included in the study. The cutoff point of breast cancer surgery for high-volume hospital and high-volume surgeon was 91 and 21 cases. Table 1 shows their baseline characteristics. The mean age at diagnosis differed by individual socioeconomic status. The mean age was 57 years in low-SES patients, 51 years in moderate-SES patients, and 48 years in high-SES patients. 

**Table 1:** Baseline characteristics (*n*=5750).

	Low SES			Moderate SES			High SES		
Variable	(*n*=2082)			(n=2292)			(n=1376)		p value
	*n*	%		n	%		n	%	
Patient age, mean±SD	57±13			51±11			48±8		<0.001*
Patient age group, years									<0.001
< 45	395	19.0		611	26.7		506	36.8	
45-54.9	520	25.0		990	43.2		585	42.5	
55-64.9	542	26.0		442	19.3		254	18.5	
65-74.9	453	21.8		181	7.9		29	2.1	
75 and older	172	8.3		68	3.0		2	0.1	
CCIS									<0.001
0	1353	65.0		1498	65.0		1011	73.5	
≥1	729	35.0		794	34.6		365	26.5	
Geographic region									<0.001
Northern	1275	61.2		1123	49.0		962	69.9	
Central	367	17.6		462	20.2		189	13.7	
Southern	415	19.9		668	29.1		198	14.4	
Eastern	25	1.2		39	1.7		27	2.0	
Urbanization level									<0.001
Urban	778	37.4		663	28.9		650	47.2	
Suburban	1020	49.0		1015	44.3		575	41.8	
Rural	284	13.6		614	26.8		151	11.0	

*one-way ANOVA test.

### Low-volume hospital

**Figure 1:**
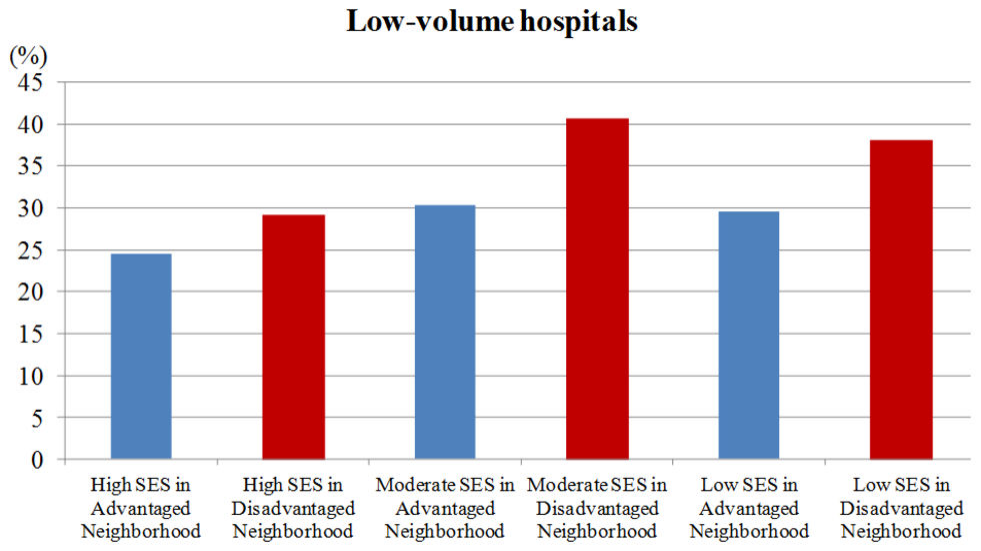
The proportion of receiving breast cancer surgery at a low-volume hospital in breast cancer patients by individual and neighborhood SES.

Breast cancer patients in disadvantaged neighborhood were more likely to visit low-volume hospitals (Figure 1). Table 2, in multivariate analysis, Model A shows patients with a moderate/low-SES in advantaged/disadvantaged neighborhood had the odds ratios ranging from 1.29 to 2.12 (p<0.05) to receive breast cancer surgery at a low-volume hospital, compared with high-SES patients in advantaged neighborhood. In Model B, after adjustment for patients' age, CCIS, geographic region and urbanization level of residence, only patients with a moderate- and low-SES in disadvantaged neighborhoods had an odds ratio of 1.47 (95% CI=1.19-1.81) and 1.31 (95% CI=1.05-1.64) respectively, compared with high-SES patients in advantaged neighborhood. Predictors of surgery at a low-volume hospital were moderate- and low-SES in disadvantaged neighborhoods, increased age, and not living in urban areas. 

**Table 2:** Odds ratios of individual SES for low-volume hospital in advantaged and disadvantaged neighborhoods.

Variable	Model A				Model B*		
	Odds ratio	95% CI	p value		Odds ratio	95% CI	p value
Neighborhood socioeconomic status							
High SES in Advantaged Neighborhood	1				1		
High SES in Disadvantaged Neighborhood	1.27	0.99-1.62	0.056		0.92	0.71-1.19	0.514
Moderate SES in Advantaged Neighborhood	1.34	1.09-1.66	0.006		1.20	0.97-1.49	0.093
Moderate SES in Disadvantaged Neighborhood	2.12	1.75-2.56	<0.001		1.47	1.19-1.81	<0.001
Low SES in Advantaged Neighborhood	1.29	1.06-1.58	0.012		1.13	0.92-1.40	0.240
Low SES in Disadvantaged Neighborhood	1.89	1.54-2.32	<0.001		1.31	1.05-1.64	0.016
Patients' age					1.01	1.01-1.02	0.002
CCIS					1.01	0.99-1.03	0.170
Geographic region							
Northern					1		
Central					1.07	0.91-1.27	0.401
Southern					1.42	1.22-1.66	<0.001
Eastern					5.87	3.59-9.61	<0.001
Urbanization level							
Urban					1		
Suburban					1.34	1.17-1.54	<0.001
Rural					1.48	1.21-1.80	<0.001
Hosmer-Lemeshow goodness-of-fit test					4.16		0.842

* Adjusted for patients' age, Charlson Comorbidity Index Score, geographic region and urbanization level of residence.

### Low-volume surgeon

**Figure 2:**
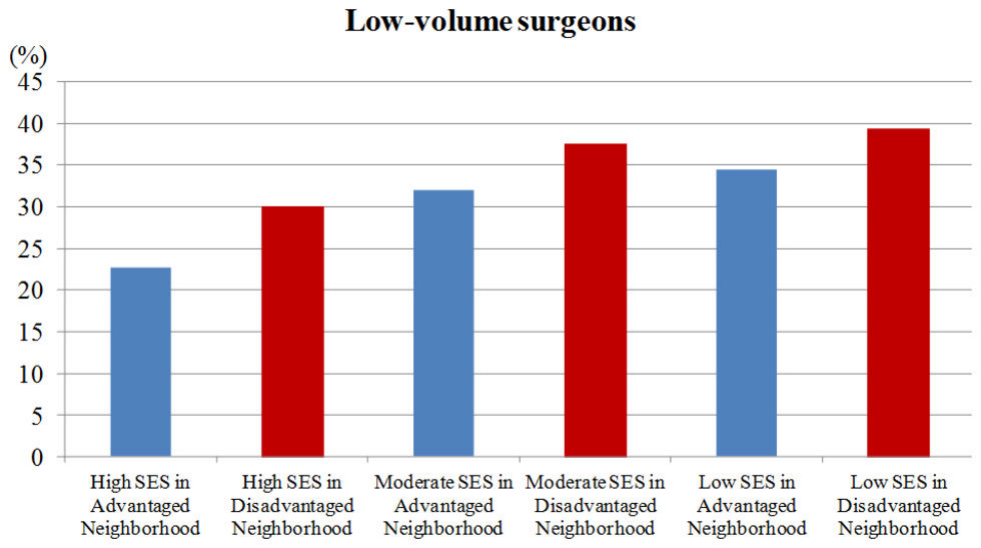
The proportion of receiving breast cancer surgery from a low-volume surgeon in breast cancer patients by individual and neighborhood SES.

Breast cancer patients with lower SES were more likely to visit low-volume surgeons (Figure 2). Table 3, in Model B multivariate analysis, after adjustment for patients' age, CCIS, geographic region and urbanization level of residence, patients with a moderate- and low-SES in advantaged/disadvantaged neighborhoods had the odds ratios ranging from 1.51 to 1.80 (p<0.001) to receive breast cancer surgery from a low-volume surgeon, compared with high-SES patients in advantaged neighborhoods. Predictors of surgery by a low-volume surgeon were moderate- and low-SES in advantaged/disadvantaged neighborhoods, and increased age.

**Table 3:** Odds ratios of individual SES for low-volume surgeons in advantaged and disadvantaged neighborhoods.

Variable	Model A				Model B*		
	Odds ratio	95% CI	p value		Odds ratio	95% CI	p value
Neighborhood socioeconomic status							
High SES in Advantaged Neighborhood	1				1		
High SES in Disadvantaged Neighborhood	1.47	1.15-1.88	0.002		1.25	0.97-1.62	0.087
Moderate SES in Advantaged Neighborhood	1.60	1.29-1.98	<0.001		1.51	1.22-1.87	<0.001
Moderate SES in Disadvantaged Neighborhood	2.05	1.69-2.49	<0.001		1.75	1.42-2.16	<0.001
Low SES in Advantaged Neighborhood	1.79	1.47-2.19	<0.001		1.55	1.26-1.91	<0.001
Low SES in Disadvantaged Neighborhood	2.21	1.80-2.72	<0.001		1.80	1.44-2.25	<0.001
Patients' age					1.01	1.01-1.02	<0.001
CCIS					1.02	0.99-1.04	0.092
Geographic region							
Northern					1		
Central					1.06	0.90-1.25	0.464
Southern					1.12	0.96-1.31	0.152
Eastern					4.64	2.90-7.44	<0.001
Urbanization level							
Urban					1		
Suburban					1.15	1.01-1.32	0.047
Rural					1.01	0.82-1.23	0.956
Hosmer-Lemeshow goodness-of-fit test					7.88		0.445

* Adjusted for patients' age, Charlson Comorbidity Index Score, geographic region and urbanization level of residence.

In Hosmer-Lemeshow goodness-of-fit test for low-volume hospital and low-volume surgeon, both p values >0.05 that shows the model has a good fit to the data.

Table 4 shows patients in disadvantaged neighborhoods had lower healthcare resources, such as physicians per 10,000 residents and pharmacists. High-SES patients in advantaged neighborhoods were more likely to have higher median household income. This data supported the reason for using six individual and neighborhood SES groups. 

**Table 4:** Sociodemographic characteristics by individual and neighborhood socioeconomic status (*n*=5750).

	High individual SES			Moderate individual SES			Low individual SES		
	Advantaged neighborhood	Disadvantaged neighborhood		Advantaged neighborhood	Disadvantaged neighborhood		Advantaged neighborhood	Disadvantaged neighborhood	p value
Number of patients	858	518		891	1401		1176	906	
Mean age, mean±SD	47±8	49±8		49±9	52±11		58±13	56±13	<0.001
Education ≥ high school, %	98.0	87.5		96.4	74.7		98.1	86.0	<0.001
Median household income, NT$1000	647±76	507±35		615±58	494±41		628±68	502±35	<0.001
Health care-related resources									
Physicians per 10,000 persons, mean±SD	28±23	17±17		23±17	13±14		26±21	13±14	<0.001
Pharmacists per 10,000 persons, mean±SD	11±13	5±4		8±10	4±4		10±12	4±4	<0.001

## Discussion

From a national database study of 5,750 patients who received breast cancer surgery under a nationwide universal health insurance system in 2006, we explored the association of the combined effect of individual and neighborhood SES on the access to a low-volume provider for surgical treatment in cases of breast cancer. Our series revealed that breast cancer patients with moderate/low SES in disadvantaged neighborhoods were more likely to receive breast cancer surgery at low-volume hospitals, while individuals with moderate/low SES were more likely to receive breast cancer surgery from low-volume surgeons. Even under a nationwide universal health insurance system, disparities in access to healthcare exist. The authorities may provide more breast cancer treatment information to these vulnerable groups and the public health policies should keep focusing on them.

The strengths of our study are based on the fact that it was a large population-based cross-sectional study (n=5,750), with nearly complete follow-up information of access to healthcare institutes among the whole study population (99%), as well as the fact that the dataset is routinely monitored for diagnostic accuracy by the National Health Insurance Bureau of Taiwan. The trend in access to a low-volume provider among the surgeons and hospitals showed a positive association between combined SES and low-volume providers. 

The SES of a patient played a key factor in receiving breast cancer surgery. Inequalities in the utilization of medical resources may affect patients from different SES. High-SES breast cancer patients, either living in advantaged or disadvantaged neighborhoods, have a lower rate of visiting a low-volume provider. High-SES breast cancer patients may use their knowledge, information, money, social connections, and other available resources to improve their health status even they live in disadvantaged neighborhoods. On the contrary, lower-SES patients even in advantaged neighborhoods where possessed more health-related resources were associated with higher odds to receiving breast cancer surgery from low-volume surgeons. A lower SES may cause stress, depression, and isolation on patients [10], which made it harder for lower-SES patients to obtain useful information or advice from relatives, friends, or acquaintances. 

Our previous study showed that low-SES breast cancer patients had a higher risk of mortality [11]. In the current study, we found such patients were more likely to be treated by low-volume providers, a factor for survival disparities [2,12–14] this correlation may be one of the reasons for the survival disparities across breast cancer patients with different SES. 

Our findings can be applied to policy intervention to reduce healthcare disparities. It has been suggested that the referral of patients to high-volume hospitals will provide a better application of recommended processes of care [13,15]. Our study showed that in addition to referring breast cancer patients to a high-volume hospital, referring patients to a high-volume surgeon may be another consideration. 

However, under a universal insurance coverage lower-SES patients in disadvantaged neighborhoods were still more likely to receive breast cancer surgery at a low-volume hospital. Inconvenience and a considerable amount of expenses to traveling for surgery may be one of the reasons. Therefore, a more important point for lower-SES breast cancer patients other than referring them to a high-volume provider is the difference in their treatment quality comparing to high-SES patients. Research organizations and payers may conduct clinical quality improvement research to identify care, treatment strategies, and process differences between providers of different volumes. It has been suggested that there are some low-volume providers who get good results, and therefore referral to relative low-volume providers should be supported if good outcomes can be demonstrated [15]. This may be more convenient for older and lower-SES breast cancer patients to be treated close to home and may be less controversial than referring every patient to high-volume providers without considerations of their age, lower-SES and geographical factors. 

Our study has limitations. First, our database does not contain address information that the distance from each patient residence to the nearest high-volume hospital and surgeon cannot be obtained. But we collected the neighborhood health care-related resources as a reference of their nearest access to medical service providers. Second, the diagnosis of breast cancer, and any comorbidity, was completely dependent on ICD codes. Nonetheless, the National Health Insurance Bureau of Taiwan randomly reviews the charts and interviews patients in order to verify diagnosis accuracy. Third, the relationship of the stages of breast cancer patients could not be assessed because cancer stage data was not included in the database. However, Begg et al. revealed that cancer stage was independent of caseload volume in a SEER-Medicare linked database [16]. 

In summary, even under a nationwide universal health insurance system, our findings indicate that disparities in access to healthcare exist. Breast cancer patients from a lower individual and neighborhood SES are more likely to receive breast cancer surgery from low-volume providers. The authorities should provide more breast cancer treatment information to these vulnerable groups and help these groups from receiving inadequate treatment. The public health policies should keep focusing on lower SES groups to reduce health disparities.

## Supporting Information

Appendix S1 **The process of defining the hospital and the surgeon volume.** Table S1, defining the category of hospital volume. Table S2, defining the category of surgeon volume.
(DOC)
