# Supplementary material for: The Association of Socioeconomic Status and Access to Low-Volume Service Providers in Breast Cancer
Source: PLoS One. 2013 Dec 2;8(12):e81801. doi: 10.1371/journal.pone.0081801 (PMC3846901; doi:10.1371/journal.pone.0081801)
Supplement: Appendix S1 — The process of defining the hospital and the surgeon volume. Table S1, defining the category of hospital volume. Table S2, defining the category of surgeon volume. (DOC) [file pone.0081801.s001.doc]

**Supporting Information**

**Appendix S1**

1. The caseload of each hospital and surgeon was calculated.

2. Sorting the hospital’s and surgeon’s order by caseload number.

3. Defining the category of caseload was as the following steps.

Table S1. The process of defining the category of hospital volume.

| Hospital ID | Caseload | Cumulative case |
| --- | --- | --- |
| 1 | 1 | 1 |
| 2 | 1 | 2  Roughly  1/3 cases of  breast cancer patients |
| 3 | 2 | 4  Low volume |
| 4 | 2 | 6 |
| . |  |  |
| . |  |  |
| 121 | 87 | 1979 |
| 122 | 91 |  |
| 123 | 97 | Roughly  1/3 cases of  breast cancer patients |
|  |  | Medium volume |
|  |  |  |
| 132 | 216 | 3864 |
| 133 | 217 |  |
|  |  | Roughly  1/3 cases of  breast cancer patients |
|  |  | High volume |
| . | . |  |
| 137 | . |  |
| 138 | 456 | 5750 |

Table S2. The process of defining the category of surgeon volume.

| Surgeon ID | Caseload | Cumulative case |
| --- | --- | --- |
| 1 | 1 | 1 |
| 2 | 1 | 2  Roughly  1/3 cases of  breast cancer patients |
| 3 | 2 | 4  Low volume |
| 4 | 2 | 6 |
| . |  |  |
| . |  |  |
| 485 | 20 | 1905 |
| 486 | 21 |  |
| 487 |  | Roughly  1/3 cases of  breast cancer patients |
|  |  | Medium volume |
|  |  |  |
| 541 | 80 | 3890 |
| 542 | 88 |  |
|  |  | Roughly  1/3 cases of  breast cancer patients |
|  |  | High volume |
| . | . |  |
| 553 | . |  |
| 554 | 217 | 5750 |
